# Laparoscopic repair of strangulated Morgagni hernia

**DOI:** 10.1186/1749-7922-2-27

**Published:** 2007-10-12

**Authors:** Michael D Kelly

**Affiliations:** 1 Department of Upper GI Surgery, Frenchay Hospital, Bristol, BS16 1LE, UK

## Abstract

A 73 year old man presented with vomiting and pain due to a strangulated Morgagni hernia containing a gastric volvulus. Laparoscopic operation allowed reduction of the contents, excision of necrotic omentum and the sac, with mesh closure of the large defect. A brief review of the condition is presented along with discussion of the technique used.

## Introduction

Morgagni hernia is a rare diaphragmatic hernia that develops through a congenital retrosternal defect. In adults they are generally asymptomatic and are found incidentally during laparoscopy or imaging for another condition. However, they may present as an emergency with abdominal pain due to strangulation of the hernia contents. An unusual case is reported of a strangulated Morgagni hernia, which presented with vomiting and abdominal pain due to a gastric volvulus.

## Case presentation

**Figure 1:**
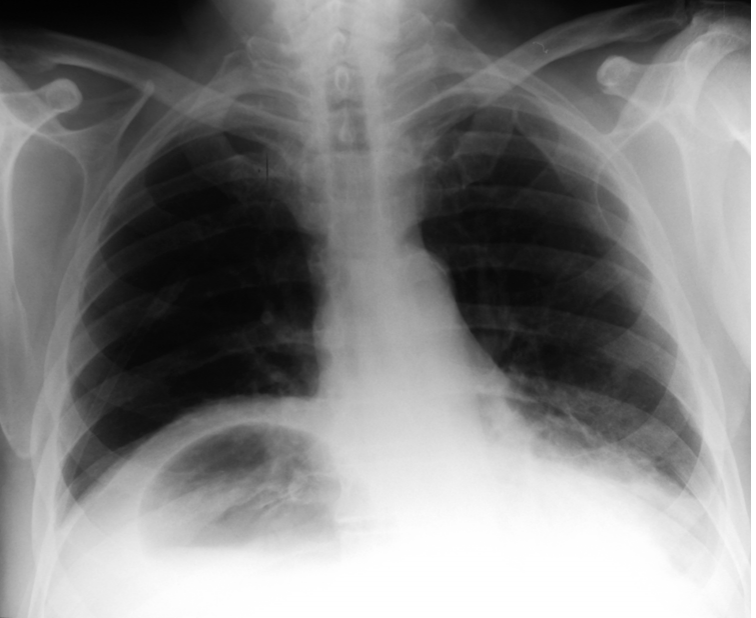
Chest x-ray on admission showing an unusual air fluid level in the lower chest.

**Figure 2:**
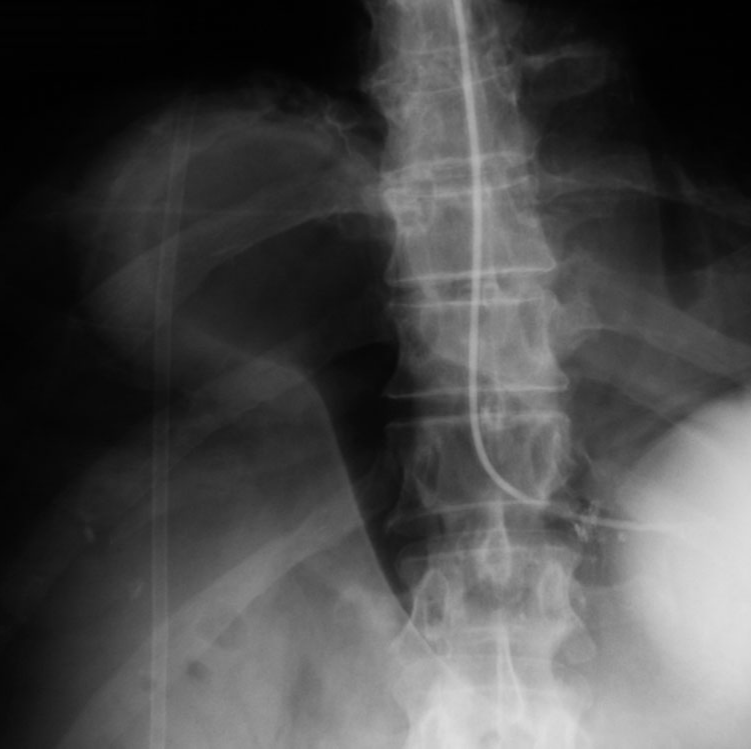
Plain x-ray showing the gastric volvulus.

**Figure 3:**
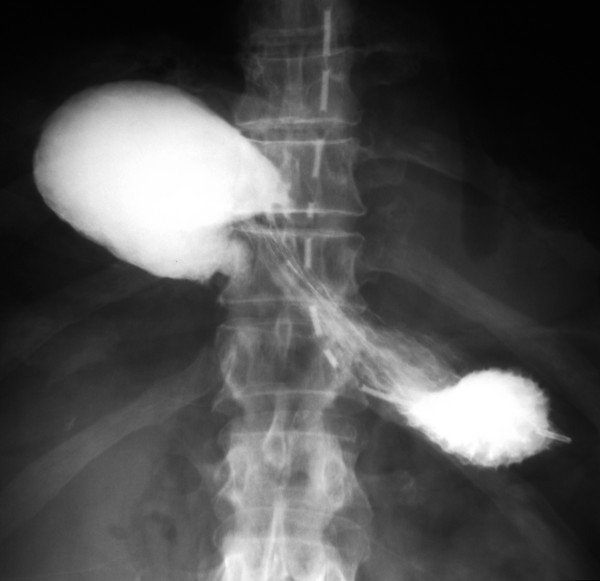
Upper GI contrast study via a gastric tube showing gastric volvulus and obstruction.

A 73-year-old man presented with a 24-hour history of vomiting and severe, constant epigastric pain. In the preceding three months he had noticed intermittent, dull epigastric pain worse on lying flat. He suffered from type II diabetes, hypertension, asthma and intermittent claudication. He had previously undergone treatment for a transitional cell carcinoma (TCC) of the bladder and repair of umbilical and inguinal hernias. He was a retired electrician with previous asbestos exposure and he was an ex-smoker. On examination, he was tachycardic and pyrexial with a distended, generally tender abdomen but no signs of peritonitis. Respiratory examination was unremarkable and his blood tests showed a raised white cell count (16.1 × 10^9^/L) along with raised serum creatinine (189 μmol/L) and urea (11.9 mmol/L). Chest x-ray showed an unusual air fluid level in the lower chest (fig 1). Under fluoroscopy, a fine bore tube was inserted and a gastrografin^® ^(Schering AG, Germany) study was done (fig 2, 3). This showed a gastric volvulus with complete obstruction within a Morgagni hernia.

**Figure 4:**
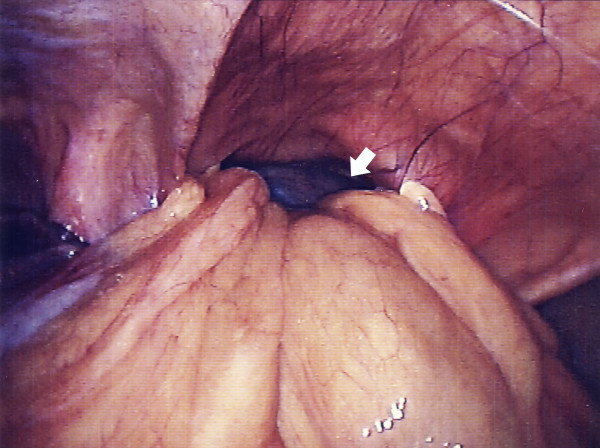
Laparoscopic view showing the Morgagni hernia. The arrow points to the ischaemic stomach, which is just visible.

**Figure 5:**
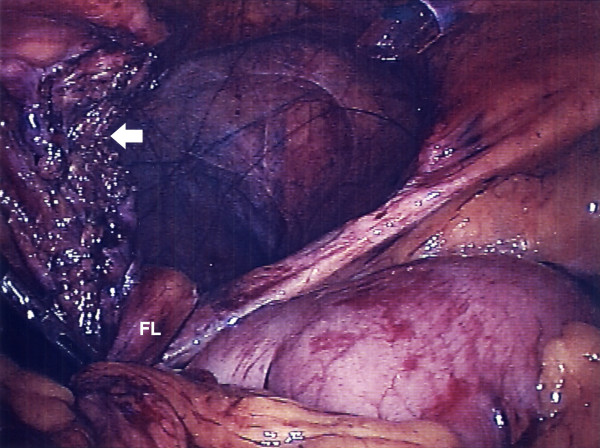
Appearance following reduction of the hernia contents. The arrow points to the site of incision of the neck of the hernia. The thin, transparent peritoneal sac can be seen lining the cavity. The falciform ligament (FL) is seen running into the defect.

**Figure 6:**
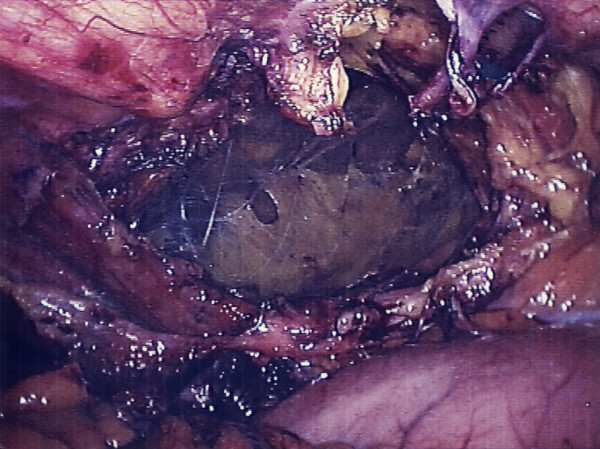
Appearance following removal of the sac and clearing of the edges of the defect in preparation for mesh placement.

**Figure 7:**
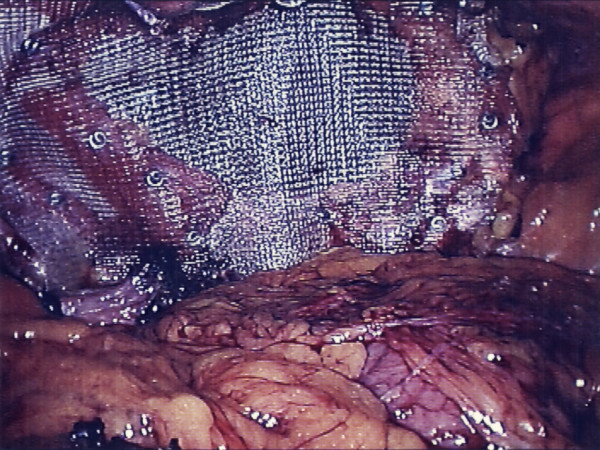
View showing the polypropelene mesh tacked in place.

An emergency laparoscopy was done via open insertion of a 10 mm port at the umbilicus and subsequent insertion of two 5 mm ports. The stomach, transverse colon and omentum were trapped in the hernia (fig 4). They were reduced with difficulty after incising the neck of the sac and the edge of the diaphragmatic defect (fig 5). There was a large section of necrotic omentum, which was resected and placed into a retrieval bag and subsequently removed. The transverse colon and stomach were carefully examined and found to be viable. The falciform ligament formed part of the wall of the sac and this was reduced along with the sac and excised (fig 6). The edges of the defect were widely cleared of peritoneum and fat to expose the muscle and fascia of the diaphragm. The defect was large and was not suitable for primary closure. Polypropelene mesh was used, with approximately 2 cm of overlap over the edges of the defect and was secured in place using 5 mm titanium tacks (Protack, Autosuture, Tyco Healthcare USA) (fig 7). The patient recovered well and was eating normally by day three although discharge was delayed due to a lower respiratory tract infection.

He presented jaundiced six weeks later and an ERCP was done to treat a bile duct stone. Unfortunately two months later he presented with shortness of breath and was diagnosed with left pleural mesothelioma from which he subsequently died. There was no evidence of the mesothelioma on either the preoperative chest xray or at the time of operation (although the pleural cavities were not opened). Interestingly, on review of his medical records he had been noted to have a small Morgagni hernia on routine chest x-ray 21 years before however the patient was not informed and no action had been taken.

## Discussion

Morgagni, an Italian anatomist, described in 1769 a hernia through a defect in the diaphragm immediately behind the sternum [1]. The hernial sac most commonly extends to the right and if large enough may contain colon, omentum and stomach. The defect lies between the sternal and anterior costal fibres of the diaphragm and is reported to represent around 3% of congenital diaphragmatic hernias [1]. It may present in the newborn with respiratory distress however most are not diagnosed until later in life.

In the adult, Morgagni hernias are generally asymptomatic being an incidental finding on imaging or laparoscopy [1,2]. Some authors recommend repair even in asymptomatic people to avoid the risk of strangulation and emergency surgery [2,3]. If there were to be radiological evidence of incarcerated tissue then elective operation should be recommended. With the advent of effective laparoscopic repair it is likely that more asymptomatic patients will come to operation.

Morgagni hernias may become symptomatic and various upper GI complaints have been attributed to this type of hernia, including indigestion and flatulence. Rarely, the hernia may present acutely when its contents become strangulated and emergency operation will be necessary.

Laparoscopic repair of Morgagni hernia was first described by Kuster and colleagues in 1992 and has rapidly replaced open transabdominal or transthoracic approaches although most emergency cases are still managed by open operation [4,1]. There is still some debate as to the merits of resecting the sac and whether primary closure of the defect is preferable to mesh placement. In the case detailed herein, the defect was large and primary closure was not feasible. Polypropelene mesh was carefully tacked in place with a wide overlap onto fascia. This is easily done and is likely to form a strong repair with tissue ingrowth into the mesh fixing it in place. Due to the position of the defect, which will be covered by stomach and omentum, it is unlikely that bowel would become adherent and the use of special mesh was not considered necessary. Fixation of the mesh may be achieved by sutures or tacks, although if tacks are used, care must be taken as there is a report of postoperative cardiac tamponade due to bleeding from an epicardial artery [5]. Unfortunately no significant followup was possible on this patient and further reports will be needed to confirm the durability of this technique.

In hiatal hernia, the esophagus forms part of the wall of the sac. The sac should be dissected and reduced to allow the esophagus to be encircled and mobilised for crural dissection and repair, and to ensure an adequate length of intraabdominal esophagus. However, in a Morgagni hernia the sac is complete with only the falciform ligament and ligamentum teres in its wall and it may not be necessary to remove the sac. If it is to be left in place, it is important to circumcise the neck of the sac and reflect the peritoneum and fat to allow wide fixation of the mesh to muscle and aponeurosis.

It is important for clinicians to be aware of strangulated Morgagni hernia as a rare cause of the acute abdomen and prompt diagnosis will ensure a higher chance of laparoscopic repair. Strangulated cases can be treated laparoscopically even if necrotic tissue has to be resected. As the defect is anterior, it is advisable to use a 30° laparoscope with the patient in low stirrups so the surgeon can stand between the patients' legs.

While these hernias are usually considered to be benign it is logical to expect that they will enlarge with time as in the presently reported case. Caution is advised before dismissing Morgagni hernias in adults as being clinically insignificant.
